# Identification of Cancer Stem-Like Side Population Cells in Purified Primary Cultured Human Laryngeal Squamous Cell Carcinoma Epithelia

**DOI:** 10.1371/journal.pone.0065750

**Published:** 2013-06-11

**Authors:** Chun-Ping Wu, Liang Zhou, Ming Xie, Huai-Dong Du, Jie Tian, Shan Sun, Jin-Yan Li

**Affiliations:** 1 Department of Otolaryngology-Head and Neck Surgery, Eye, Ear, Nose and Throat Hospital, Fudan University, Shanghai, People’s Republic of China; 2 Central Laboratory, Eye, Ear, Nose and Throat Hospital, Fudan University, Shanghai, People’s Republic of China; 3 Institute of Biomedical Science, Stem Cell and Regenerative Medicine, Shanghai Medical College, Fudan University, Shanghai, People’s Republic of China; 4 Department of Cellular and Genetic Medicine, Shanghai Medical College, Fudan University, Shanghai, People’s Republic of China; Stony Brook University, United States of America

## Abstract

Cancer stem-like side population (SP) cells have been identified in many solid tumors; however, most of these investigations are performed using established cancer cell lines. Cancer cells in tumor tissue containing fibroblasts and many other types of cells are much more complex than any cancer cell line. Although SP cells were identified in the laryngeal squamous cell carcinoma (LSCC) cell line Hep-2 in our pilot study, it is unknown whether the LSCC tissue contains SP cells. In this study, LSCC cells (LSCCs) were primary cultured and purified from a surgically resected LSCC specimen derived from a well-differentiated epiglottic neoplasm of a Chinese male. This was followed by the verification of epithelium-specific characteristics, such as ultrastructure and biomarkers. A distinct SP subpopulation (4.45±1.07%) was isolated by Hoechst 33342 efflux analysis from cultured LSCCs by using a flow cytometer. Cancer stem cell (CSC)-associated assays, including expression of self-renewal and CSC marker genes, proliferation, differentiation, spheroid formation, chemotherapy resistance, and tumorigenicity were then conducted between SP and non-SP (NSP) LSCCs. *In vitro* and *in vivo* assays revealed that SP cells manifested preferential expression of self-renewal and CSC marker genes, higher capacity for proliferation, differentiation, and spheroid formation; enhanced resistance to chemotherapy; and greater xenograft tumorigenicity in immunodeficient mice compared with NSP cells. These findings suggest that the primary cultured and purified LSCCs contain cancer stem-like SP cells, which may serve as a valuable model for CSC research in LSCC.

## Introduction

Cancer stem-like side population (SP) cells have been successfully identified in a wide range of solid tumors, including breast cancer [1], [2], hepatocellular carcinoma [3]–[7], lung cancer [8], [9], gastrointestinal cancer [10]–[12], prostate cancer [13], gallbladder cancer [14], ovarian cancer [15], endometrial cancer [16], pancreatic cancer [17], [18], urological cancer [19], [20], glioblastoma [21], melanoma [22], osteosarcoma [23], [24], mesenchymal neoplasms [25], nasopharyngeal cancer [26], oral cancer [27], [28], and other head and neck cancers [29], [30]. However, most of these investigations have been performed using established cancer cell lines. Although established cancer cell lines are useful tools in basic and preclinical cancer research, they are simplified mimics of complex, heterogeneous, solid cancerous tissues. Cancer cells in primary tumor tissue containing fibroblasts, stroma cells, lymphocytes, and other types of cells are much more complex than the cells in any cancer cell line. Therefore, primary cultured and purified cancer cells deriving from the cancerous tissues may be a better representation of the original tumor.

Laryngeal squamous cell carcinoma (LSCC) is one of the most common malignancies of the head and neck region. In recent years, LSCC patients in the advanced stage have still tended to succumb to locoregional recurrence and distant metastasis. Cancer stem-like SP cells play a critical role in tumor initiation, maintenance, progression, and relapse [31]–[33]. Therefore, ongoing research on SP cells to develop new agents that target cancer stem cells (CSCs) is urgently needed.

Our pilot study identified cancer stem-like SP cells in the LSCC cell line Hep-2 [30]. However, it is unknown whether the LSCC solid tumor contains SP cells. In this study, for the first time, we used Hoechst 33342 efflux analysis to identify SP cells directly from purified, primary cultured, well-differentiated LSCC cells (LSCCs) derived from a Chinese male patient undergoing laryngectomy for epiglottic carcinoma. We found that the primary cultured LSCCs also contained a distinct SP subpopulation, which accounted for 4.45±1.07% of the total cancer cells. In addition, by *in vitro* and *in vivo* assays, we documented that SP cells harbored more cancer stem-like properties compared with non-SP cells (NSP).

## Materials and Methods

### Ethics Statement

Tumor specimen was obtained with the approval of the Ethics Committee of the Eye, Ear, Nose and Throat Hospital, Fudan University, Shanghai, China. Signed informed consent was obtained from the patient. The protocol was approved by the Shanghai Medical Experimental Animal Care Committee. All surgery was performed under sodium pentobarbital anesthesia, and all efforts were made to minimize suffering.

### Patient Information

The patient was an untreated 68-year-old Chinese male who underwent laryngectomy for squamous cell carcinoma deriving from the epiglottis, Stage IVa, T4aN2M0, based on the 6th edition Union for International Cancer Control (UICC) TNM classification system. Notably, he did not have a family history of head and neck cancer, but did have a 40-year history of smoking and 30-year history of alcohol use.

### Primary Culture and Purification of LSCCs

A surgically resected tumor specimen was immersed in cold triple antibiotic phosphate buffered saline (PBS) containing 1% penicillin/streptomycin and amphotericin B (10 µg/ml) (Invitrogen, Buffalo, NY, USA), and scissored into small fragments, which were then dissociated enzymatically in RPMI 1640 medium containing type IV collagenase (Sigma) at a final concentration of 200 U/ml for at least 12 h at 37°C. The cells and remaining fragments were then rinsed and suspended in BEGM™ (bronchial epithelial cell growth medium) (Catalog CC-3170; Lonza, Walkersville, MD, USA) supplemented with 1% penicillin/streptomycin. The suspension was then seeded in 60-mm petri dishes in a humidified 5% CO_2_ incubator at 37°C. After 3–4 days of incubation, some of the fragments and cells adhered to the dish; those that did not adhere were washed away before the medium was renewed. Fibroblasts were removed by brief exposure to 0.25% trypsin-EDTA (Invitrogen, Buffalo). Cancer cells near confluence were detached with 0.25% trypsin-EDTA and subcultured. Cells were stored in liquid nitrogen from passage 1.

### Morphological Examination and Immunocytochemistry

Cultured LSCCs were examined under a phase-contrast microscope for morphological characteristics. To validate the epithelial origin, LSCCs growing for 24 h on glass coverslips were fixed with 4% paraformaldehyde (PFA) for 10 min and then incubated in 1% bovine serum albumin (BSA)/10% normal goat serum/0.3% Triton X-100 (Boster, Wuhan, China) at room temperature for 40 min to block nonspecific interactions and permeabilize the cells. Monoclonal antibodies against human cytokeratin (CK) (pan) (1∶50, clone AE1/AE3; Maixin Biotech, Fuzhou, China ), cytokeratin 5 (CK5) (1∶50, catalog C0246; Anbo, San Francisco, CA, USA), and vimentin (1∶50, clone SP20; Maixin Biotech) were added and incubated overnight at 4°C, followed by the addition of secondary antibody and incubation in the dark for 1 hour at 37°C.The nuclei were stained with 4′, 6-diamidino-2-phenylindole (DAPI, Boster, Wuhan, China). The secondary antibody was Cy3-conjugated goat anti-mouse/rabbit immunoglobulin (Ig)G heavy and light chains (H+L) (1∶100; Jackson, Lancaster, PA, USA).

### Transmission Electron Microscopy

A pellet obtained from the harvested LSCCs at passage 2 was fixed with 2.5% glutaraldehyde and postfixed in 1% osmium tetroxide. The sample was dehydrated through a graded alcohol series and embedded in resin. Ultrathin sections were cut, stained with uranyl acetate and lead citrate, and observed under a Philips CM120 transmission electron microscope (TEM).

### Flow Cytometer for the Purity of Primary Cultured LSCCs

Cultured LSCCs were trypsinized, washed in PBS, fixed in 4% PFA for 10 min, and incubated in 1% BSA/10% normal goat serum/0.3% Triton X-100 (Boster) at room temperature for 40 min to permeabilize the cells and block nonspecific interactions. This was followed by incubation in PBS containing CK(pan)−fluorescein isothiocyanate antibody (1∶500, clone C-11; Sigma) for 30 min at room temperature. Then cells were washed twice with PBS and analyzed using a CyAn ADP flow cytometer (Beckman Coulter, Fullerton, CA, USA). Cells treated with PBS instead of CK(pan) served as control.

### Detection and Isolation of SP and NSP Cells using Flow Cytometry

Cultured LSCCs in exponential growth phase were rinsed in PBS, trypsinized, and suspended at a concentration of 1×10^6^ cells/ml in cold BEGM medium. Hoechst 33342 (Sigma) was added at a final concentration of 5 µg/ml in the presence or absence of verapamil (Sigma) at 50 µmol/l, preincubated for 30 min at 37°C. This was followed by a 90-min incubation in the dark at 37°C with interval shaking. After washing, 1 µg/ml propidium iodide (Sigma) was added to exclude dead cells, and samples were analyzed in an EPICS ALTRA flow cytometer (Beckman Coulter, Fullerton, CA, USA).

### Cell Viability Assay

Freshly sorted SP and NSP LSCCs were seeded in 96-well plates at a density of 5,000 cells/well in 0.2 ml of BEGM medium and serum-free medium (SFM; described in the following spheroid formation assay). Each group was repeated in 6 wells, and medium without cells served as a control. Cell viability was measured using the Cell Counting Kit-8 (CCK-8; Dojindo Laboratories, Kumamoto, Japan), following the manufacturer’s instructions. The incubation lasted 3 h, and the assay was conducted in triplicate. Ultraviolet absorbance was measured at 450 nm with an enzyme-linked immunosorbent assay (ELISA) plate reader.

### Quantitative Real-time PCR (qRT-PCR)

Trizol reagent (Invitrogen, Carlsbad, CA, USA) was used to extract total RNA from isolated SP and NSP cells using. Reverse transcription was performed using PrimeScript® RT reagent kit with gDNA eraser (Takara, Dalian, China). Real-time PCR was done with the SYBR® Premix Ex TaqTM kit (Takara) in a Lightcycler 480 instrument (Roche Diagnostics, Rotkreuz, Switzerland). Thermal cycling included an initial denaturation at 95°C for 30 s, followed by 40 cycles at 95°C for 5 s and 60°C for 30 s. Beta-actin (ACTB) was used as an endogenous control. The formula 2^−ΔΔCT^ was used to calculate the relative mRNA expression of SP versus NSP cells. The primers used for amplification are listed in Table 1.

**Table 1:** Sequences and chromosomal location of primers used for quantitative real-time PCR.

Gene	Primer Sequence (5′→3′)	Accession No.	Size (bp)	Location
ABCG2	(forward) CATCAGCGGATACTACAGA	NM_001257386	180	4q22
	(reverse) GAATAAGCCACCATCATAAGG			
BMI1	(forward) AATGCTGGAGAACTGGAA	NM_005180	125	10p11.23
	(reverse) AACTGTGGATGAGGAGAC			
OCT4	(forward) GTATTCAGCCAAACGACCAT	NM_001173531	100	6p21.31
	(reverse) CTTCCTCCACCCACTTCT			
SOX2	(forward) TGTCAAGGCAGAGAAGAG	NM_003106	223	3q26.3-q27
	(reverse) AGAGGCAAACTGGAATCA			
NANOG	(forward) CTATAACTGTGGAGAGGAAT	NM_024865	124	12p13.31
	(reverse) AGTGGTCTGCTGTATTAC			
CD44	(forward) GTTAAGTGCCTGGGGAGTCC	NM_000610	150	11p13
	(reverse) GCCACAAAGGACTTGCCAAG			
CD24	(forward) AACAGCCAGTCTCTTCGTGG	NM_013230	117	6q21
	(reverse) AGACGCCATTTGGATTGGGT			
CD133	(forward) TGCTGCTTGTGGAATAGA	NM_001145847	255	4p15.32
	(reverse) CCTGTGCGTTGAAGTATC			
ACTB	(forward) TGACGTGGACATCCGCAAAG	NM_001101	205	7p22
	(reverse) CTGGAA GTGGACAGCGAGG			

### Western Blot for CSC Markers

About 30 µg of protein samples from cell lysates of sorted SP and NSP cells were analyzed by using sodium dodecyl sulfate polyacrylamide gel electrophoresis (Beyotime, Shanghai, China), electrotransferred to polyvinylidene fluoride membranes (Millipore, Billerica, MA, USA), and probed overnight with primary antibody (Epitomics, Burlingame, CA, USA) for CD44 (1∶1,000, 1998-1), CD24 (1∶500, T3445), CD133 (1∶1,000, 3621-1), ABCG2 (1∶1,000, 3765-1), BMI1(1∶10,000, 5590-1), OCT4 (1∶1,000, 2876-1), SOX2 (1∶1,000, 2683-1), and NANOG (1∶1,000, 3369-1). Goat anti-rabbit IgG (H+L) (1∶5,000, Jackson) was then applied, followed by signal detection using BeyoECL Plus (Beyotime, Shanghai, China). ACTB antibody (1∶5,000, R1207-1; HuaAn Biotech, Hangzhou, China) was used to normalize the amount of sample loaded.

### Proliferation Assay

Sorted SP and NSP LSCCs were seeded as described above. On days 1, 3, 5, and 7 after sorting, cell growth was assayed using the CCK-8, following the manufacturer’s instructions. The incubation lasted 3 h, and the assay was conducted in triplicate. Ultraviolet absorbance was measured at 450.

### Differentiation Assay

Isolated SP and NSP cells were cultured in BEGM medium for 18 days during which the samples were restained by Hoechst 33342 on day 6, 12, and 18 to quantify the percentage of SP cells in each group. We used a flow cytometer for the analysis.

### Spheroid Formation Assay

Sorted SP and NSP cells (5,000 cells/ml) were cultured using the StemPro_ NSC SFM kit (A10509-01; Invitrogen, Carlsbad), with SFM containing Dulbecco's Modified Eagle Medium (DMEM)/F12, 20 ng/ml fibroblast growth factor, and 20 ng/ml epidermal growth factor. In addition, 1% 200 mmol/l L-glutamine (25030; Invitrogen, Grand Island, NY, USA) was added. After seeding, the spheroid formation ability of the two subsets was observed over time.

### Drug Sensitivity Assay

Sorted SP and NSP LSCCs were seeded at 5×10^3^ cells/well in 96-well plates and cultured in BEGM medium containing cisplatin (Pharmaceutical Factory, Nanjing, China) in a concentration gradient (0, 1, 3, 5, 7, 9, 11 µg/ml). Each concentration was repeated in 6 wells. The other 6 parallel wells were preincubated with 50 µmol/l verapamil as a chemosensitizer to cisplatin for 30 min at 37°C. A concentration of 0 µg/ml served as control. An additional 6 wells containing medium only served as a blank control. The cells, cultured for 48 h, were assessed by incubating with CCK-8 for 3 h. The inhibition rate (IR) was evaluated using the formula IR = 100%–survival rate (SR), and the SR was measured using the formula SR = (mean absorbance of the test wells/mean absorbance of the control wells) ×100%.

### Xenograft Tumorigenicity Assay *in vivo*

Male nonobese diabetic (NOD)/severe combined immunodeficiency (SCID) mice, 6–8 weeks of age (Slac Laboratory Animal Company, Shanghai, China), were fed in laminar flow cabinets under specific pathogen-free conditions. Sorted SP, NSP cells, and parent LSCCs without sorting were suspended in 0.2 ml of PBS and injected into the armpit of each mouse. Mice were palpated twice each week for tumor formation and were euthanized 6 weeks later. All tumor nodules were photographed, weighed, and confirmed by hematoxylin and eosin staining. P values were calculated according to the weights of SP tumors relative to those of NSP tumors.

### Statistical Analysis

Data were presented as mean±standard deviation (SD) of at least 3 independent experiments. The Student’s t or *Mann-Whitney* test was used with GraphPad Prism software version 5.00 for Windows (GraphPad Software, San Diego CA, USA) to examine the differences. Values of P<0.05 were considered significant.

## Results

### Morphological, Immunocytochemical, and Ultrastructural Features

**Figure 1:**
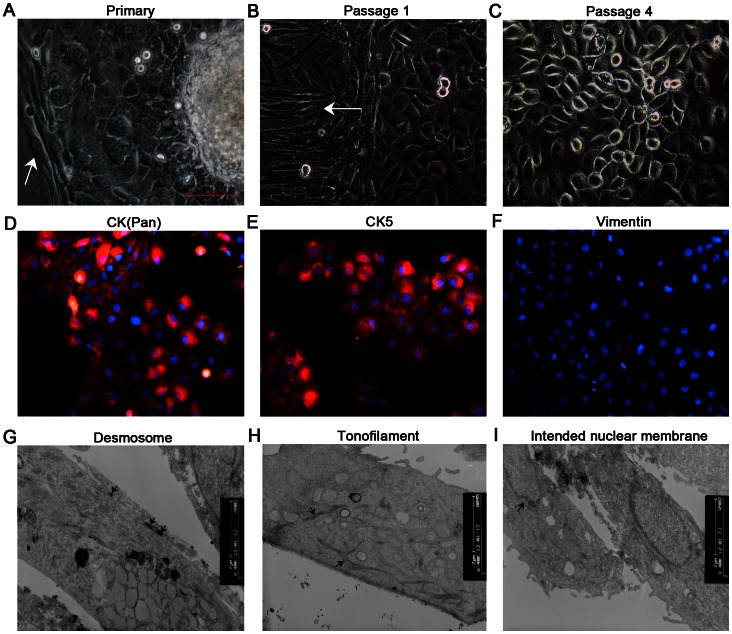
Phase-contrast photomicrograph (×200) and transmission electron micrograph (×8,400) of cultured LSCCs. (A) Laryngeal squamous cell carcinoma cells (LSCCs) grew directly from the explant 48 h after seeding, surrounded by fibroblasts (arrow). (B) At passage 1, LSCCs grew with coexisting fibroblasts (arrow). (C) At passage 4, LSCCs grew vigorously without fibroblasts and exhibited typical characteristics of malignant change, including numerous mitotic figures, a large nuclear to cytoplasmic ratio, prominent multiple nucleoli, occasional multinucleated giant cells, cytoplasmic vacuoles, round and luminescent cells, and nuclear abnormality. Positive staining of cytokeratin (CK) (pan) (D) and CK5 (E), and negative staining of vimentin (F). Ultrastructural features (arrows) of LSCCs showing desmosomes in the intercellular connections (G), tonofilaments in the cytoplasm (H), and indented nuclear membrane (I).

The cultured LSCCs grew as a monolayer in a cobblestone pattern, demonstrating their epithelial origin. Moreover, they exhibited typical characteristics of malignant change (Fig. 1A–C). Positive staining of CK(pan) and CK5 and negative staining of vimentin confirmed their epithelial lineage (Fig. 1D–F). The transmission electron micrograph revealed typical epithelial characteristics of LSCCs, including many mitochondria, rough endoplasmic reticuli, ribosomes, large irregular nuclei with the nuclear membrane showing deep indentation, and microvillus-like projections on the cell surface. Bundles of tonofilaments in the cytoplasm and numerous desmosomes in the intercellular connections were frequently observed (Fig. 1G–I).

### Purity and Sorting of SP Cells in Purified Primary Cultured LSCCs

**Figure 2:**
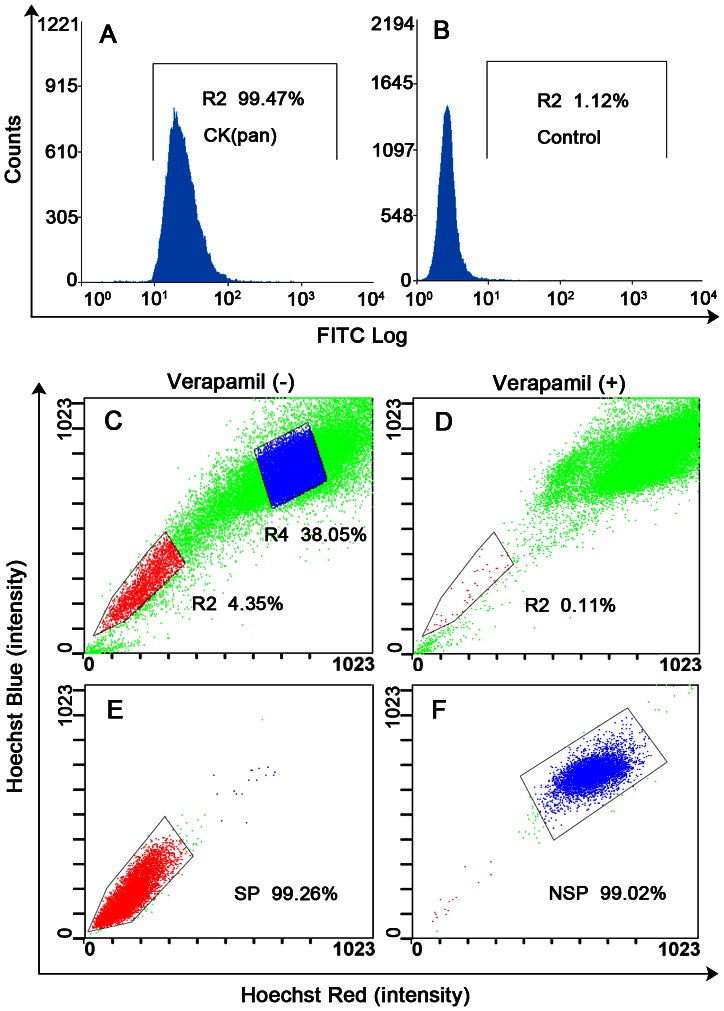
Purity of cultured LSCCs, sorting of SP and NSP cells, and purity of sorted SP and NSP cells. (A) Purity of cultured LSCCs determined by flow cytometer using cytokeratin (CK) (pan) (98.32±0.93%) and (B) control. (C) Sorting of LSCCs using Hoechst 33342. The R2 gate represents the side population (SP) cells (4.45±1.07% of total cells) and the R4 gate is the non-SP (NSP) cells. (D) The SP proportion after verapamil treatment was 0.14±0.13%. The purity of the freshly sorted SP (99.25±0.23%) (E) and NSP cells (98.98±0.22%) (F).

The purity of primary cultured LSCCs determined by flow cytometry was 98.32±0.93% (Fig. 2A,B). Cell sorting was performed after excluding dead cells and cellular debris based on scatter signals and propidium iodide fluorescence. The R2 gate showed the SP cells that were Hoechst 33342 negative/dim, and the R4 gate indicated the NSP cells that were Hoechst 33342 positive. SP cells occupied some 4.45±1.07% of the total cells. When preincubated with verapamil for 30 min, the percentage of SP cells shrank to 0.14±0.13% of the total cells (Fig. 2C,D). SP and NSP cells were collected for subsequent experiments. The purity of freshly sorted SP and non-SP cells was 99.25±0.23% and 98.98±0.22%, respectively (Fig. 2E,F).

### Cell Viability After Sorting

**Figure 3:**
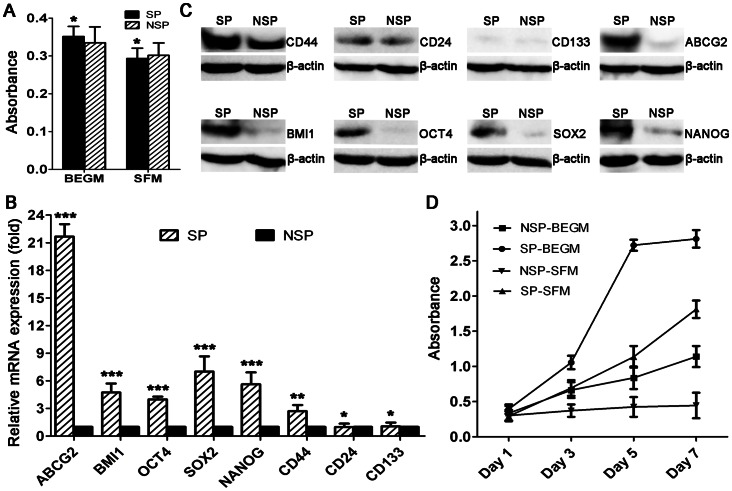
Cell viability, mRNA and protein levels of self-renewal and CSC marker genes, and cell growth rate . (A) Cell viability after sorting. No significant difference in cell viability was detected between side population (SP) and non-SP (NSP) cells in bronchial epithelial cell growth medium (BEGM) and serum-free medium (SFM). Relative (B) mRNA and (C) protein levels of self-renewal and CSC marker genes in SP and NSP cells. (D) Cell growth rate of SP and NSP cells in both BEGM and SFM determined by Cell Counting Kit-8 (CCK-8) (***P<0.01, **P<0.05, *P>0.05).

No significant differences in cell viability were detected between the freshly sorted SP and NSP LSCCs maintained in both BEGM and SFM medium (P>0.05) (Fig. 3A), indicating that the Hoechst concentration (5 µg/ml) used in this study did not potentially alter the cell viability of NSP LSCCs.

### Expression of Self-renewal and CSC Marker Genes in mRNA Level

The relative mRNA expressions of ABCG2, BMI1, OCT4, NANOG, SOX2, CD44, CD24, and CD133 in sorted SP and NSP cells were determined by qRT-PCR. The results showed that SP cells showed higher mRNA expression in ABCG2, BMI1, OCT4, NANOG, SOX2, and CD44 compared with NSP cells. However, no significant difference of CD24 and CD133 mRNA expression was detected between the SP and NSP cells (Fig. 3B).

### Protein Levels of CSC Markers

SP cells were found to have increased protein levels of CD44, ABCG2, BMI1, OCT4, SOX2, and NANOG compared with NSP cells. No significant differences of CD24 and CD133 protein levels were detected between SP and NSP cells (Fig. 3C).

### Cell Growth Rate

On days 1, 3, 5, and 7 after sorting, absorbance of the SP and NSP cells was measured using CCK-8 to determine the growth rate. In the BEGM medium, both SP and NSP cells proliferated over time. SP cells reached an exponential growth phase 3 days after seeding and by day 7 they had reached a plateau. In contrast, NSP cells continued growing slowly until day 7 before moving into an upgrade phase (P<0.001). In the SFM, SP cells proliferated stably with a speed lower than that in the BEGM. In contrast, NSP cells hardly propagated (Fig. 3D).

### Differentiation Analysis

**Figure 4:**
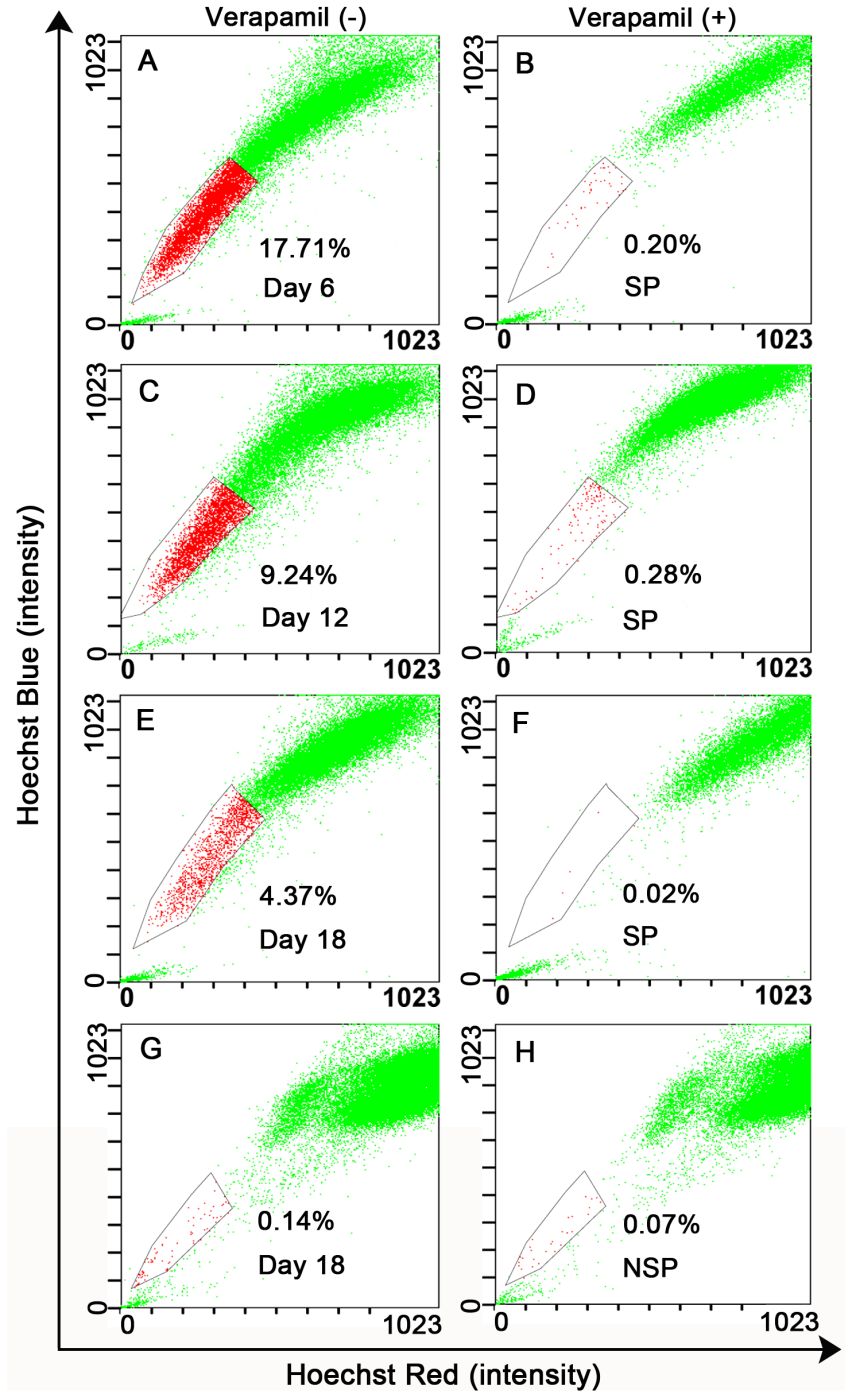
Restaining of cultured SP and NSP cells with Hoechst 33342 at different times. The percentage of side population (SP) cells detected in cultured SP cells decreased over time. The percentage of SP cells was 17.67±1.45% on day 6 (A, B), 9.08±0.61% on day 12 (C, D), and 4.45±0.63% on day 18 (E, F). In contrast, the percentage of SP cells in cultured non-SP (NSP) cells was 0.07±0.03% (G, H).

On days 6, 12, and 18 after seeding, the cultured SP and NSP cells were restained with Hoechst 33342 to measure differences in their differentiation ability. The data showed that the percentage of SP cells decreased in culture over time, at 17.67±1.45% on day 6, 9.08±0.61% on day 12, and 4.45±0.63% on day 18 (Fig. 4A–F). On day 18, the percentage of Hoechst 33342-dull/negative cells in cultured SP cells was similar to that of unsorted LSCCs, whereas cultured NSP cells only contained 0.07±0.03% SP cells (Fig. 4G–H), which may have arisen from residual SP cells from the last sorting.

### Sphere Formation

**Figure 5:**
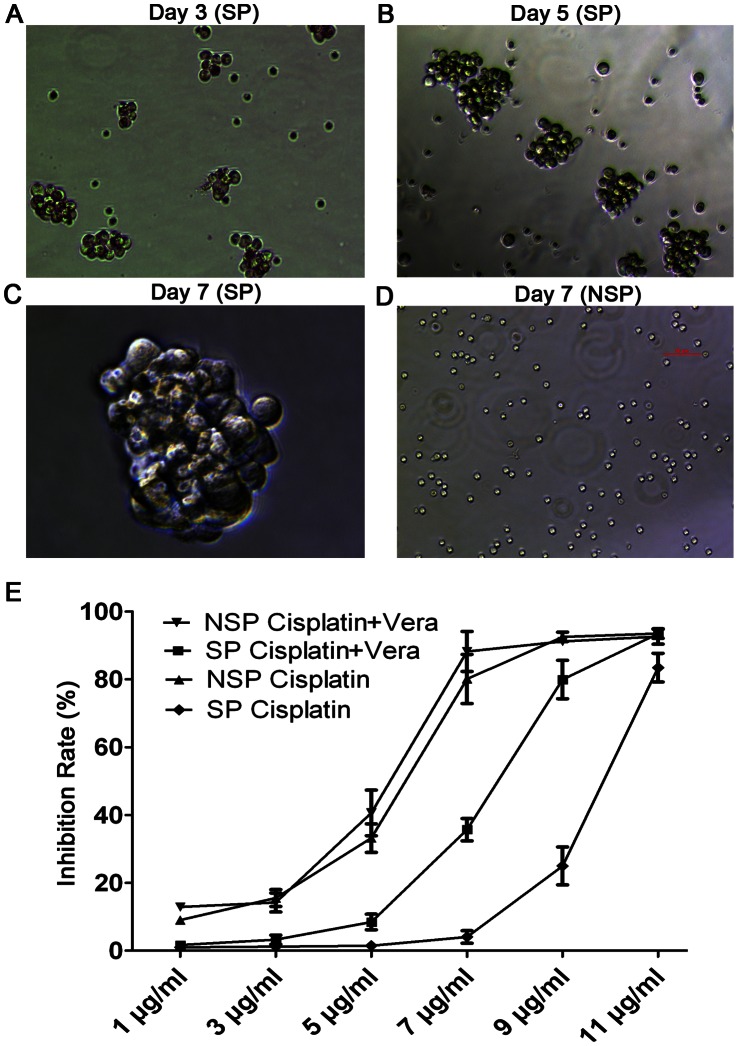
Sphere formation in serum-free medium and drug sensitivity differences between SP and NSP cells. On day 3 (A, ×100), day 5 (B, ×100), and day 7 (C, ×400), floating spherical clusters of side population (SP) cells expanded in a stepwise fashion over time. In contrast, non-SP (NSP) cells could not form spherical clusters after being cultured for 7 days (D, ×100). (E) Sensitivity of newly sorted SP and NSP to cisplatin. The inhibition rate (IR) of SP cells showed a significant difference from NSP cells at each concentration (P<0.001), with the exception of 11 µg/ml (P>0.05). SP cells were more resistant to cisplatin than NSP cells; this could be reversed by verapamil pretreatment.

Isolated SP cells proliferated stably in the stem cell culture medium. Floating spheres in suspension generated from a single SP cell of LSCCs increased in size over time (Fig. 5A–C). In contrast, some NSP cells died and others propagated very slowly and could not form visual spheres (Fig. 5D).

### Drug Sensitivity Differences Between SP and NSP Cells

Different concentrations of cisplatin were used to assess the differences of drug sensitivity between SP and NSP cells in the presence or absence of verapamil, a blocker of the cell surface ABCG2 transporter responsible for chemotherapeutic resistance. When treated with cisplatin alone, SP cells showed strong resistance until the concentration of cisplatin reached 9 µg/ml, and the IR of SP cells showed no significant difference from NSP at a concentration of 11 µg/ml (P>0.05). Compared with SP cells, the IR of NSP cells showed significant difference at each concentration (P<0.001) except at 11 µg/ml. The IR of SP cells pretreated with verapamil increased without significant difference compared to NSP cells (P>0.05), but with significant difference compared to SP cells without verapamil pretreatment (P<0.001). No remarkable changes were detected in the IR of NSP cells in the presence or absence of verapamil (P>0.05) (Fig. 5E).

### Higher Tumorigenic Capacity of SP Cells in NOD/SCID Mice

**Figure 6:**
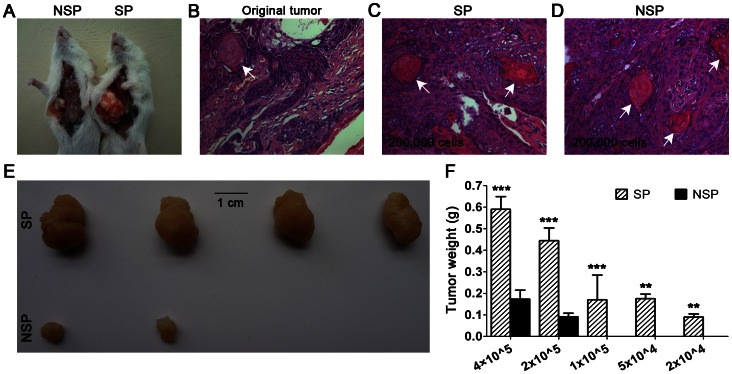
Tumor formation in NOD/SCID mice, hematoxylin and eosin staining, and statistical analysis. (A) Injection sites of nonobese diabetic (NOD)/severe combined immunodeficiency (SCID) mice. Hematoxylin and eosin staining of tumors deriving from the patient (B), mice (C, tumor formed by side population (SP); D, tumor formed by non-SP (NSP) cells). Both the original and xenografted tumors were typical, well-differentiated, squamous cell carcinoma with keratin pearls observed frequently (arrows). (E) With 2×10^5^ cells injected, SP cells formed four larger tumors (4/4), whereas NSP cells formed two much smaller tumors (2/4). (F) Statistical analysis of SP and NSP tumor weights at different injected cell quantities (***P<0.01, **P<0.05).

We used the unsorted LSCCs for the preliminary experiment and chose 4×10^5^ as the highest inoculation cell number for SP and NSP cells. With the lowest inoculation cell number (2×10^4^), SP cells formed two tumors (n = 4); in contrast, the lowest injection cell number for NSP cells to form two tumors was 2×10^5^ (n = 4). Significant differences were detected between the weights of SP tumors and NSP tumors. The pathological results confirmed that both tumors formed by SP and NSP cells were well-differentiated LSCCs, similar to the original tumor (Fig. 6; Table 2).

**Table 2:** Tumorigenicity assay of SP and NSP cells in NOD/SCID mice.

Cell number for injection (weeks)						
1×10^6^ (6)		4×10^5^ (6)	2×10^5^ (6)	1×10^5^ (6)	5×10^4^ (6)	2×10^4^ (6)
Unsorted	2/2	2/2	0/2			
SP		4/4	4/4	3/4	2/4	2/4
NSP		3/4	2/4	0/4	0/4	0/4
P value		0.000	0.001	0.002	0.010	0.048

Abbreviations: SP, side population; NSP, non-SP; NOD, nonobese diabetic; SCID, severe combined immunodeficiency. P values were calculated according to SP tumor weights versus NSP tumor weights.

## Discussion

In this study we described the successful isolation and identification of cancer stem-like SP cells directly from a surgically resected LSCC specimen derived from a well-differentiated epiglottic neoplasm in a Chinese male patient. Although primary LSCCs are difficult to culture *in vitro*, before isolation we attempted the primary culture of 40 LSCC specimens and succeeded in one case. We then purified the cultured LSCCs by repeated differential trypsinization to eliminate coexisting fibroblasts.

The monolayer and cobblestone pattern of LSCCs identified by phase-contrast microscopy, positive staining of CK(pan) and CK5, negative staining of vimentin, and the epithelial ultrastructure (including tonofilaments and desmosomes) revealed by transmission electron microscopy confirmed the epithelial lineage of the cultured LSCCs (Fig. 1).

Since the malignant squamous cells are phenotypically determined by cytokeratin, we used a flow cytometer with CK(pan) antibody to assess the purity of cultured LSCCs. Although the results should be 100%, the purity was 98.32±0.93%, which might be a result of the imperfect efficacy of the antibody (Fig. 2A,B). We then demonstrated that the purified primary cultured LSCCs contained a distinct SP subpopulation (4.45±1.07%), based on their capacity to exclude the Hoescht 33342 dye, and this capacity could be blocked by verapamil, consistent with previous reports that Hoechst 33342 exclusion is verapamil sensitive (Fig. 2C–F).

As a DNA-binding dye, Hoechst is toxic to cells, particularly at high concentrations. In order to eliminate the concern that NSP cells preferentially retain cytotoxic Hoechst 33342, which could alter the proliferation capacity, a cell viability assay was performed after sorting. Results revealed no significant differences in cell viability between the freshly sorted SP and NSP cells (P>0.05) (Fig. 3A), indicating that the cell viability of NSP LSCCs was not potentially affected by 5 µg/ml Hoechst, a concentration widely used in SP cell research [34].

In comparison to NSP cells, SP cells have increased expression of genes, such as OCT4, NANOG, BMI-1, ABCG2, and SOX2 [31], involved in the regulation of stem cell self-renewal. Interestingly, these five genes also function as CSC markers in solid tumors [35], [36]. In addition, CD44, CD24, and CD133 are well-known CSC markers in solid tumors [37]. We investigated the expression of the eight genes in both mRNA and protein levels. Consistent with previous reports, SP cells were found to have significantly upregulated expression of OCT4, NANOG, BMI-1, ABCG2, SOX2, and CD44 in both mRNA and protein levels compared with NSP cells. Of note, NSP cells also exhibited expression of CD44 protein, consistent with previously reports that CD44 is expressed in almost all cancer cells; this reflects the ambiguity of CD44 in CSC maintenance [38]. SP cells were also found to have similar expression of CD24 and CD133 in both mRNA and protein levels compared with NSP cells, consistent with previous reports that CSC phenotypes for solid tumors may not necessarily be uniform between different cancer types or even tumors of the same histological subtype (Fig. 3B,C) [37].

Self-renewal and differentiation are key properties of stem cells that allow them to give rise to progenies, including CSCs, which harbor unlimited proliferative potential, and to phenotypically diverse cells that form the bulk of the tumor. In the proliferation assay, SP cells grew significantly faster than NSP cells in both BEGM and SFM medium (Fig. 3D), confirming the unlimited proliferative potential of SP cells. Asymmetry is a particular type of cell division with an attractive mechanism in CSC because it only requires one division for both self-renewal and differentiation [39]. In the differentiation assay, the percentage of SP cells from cultured SP cells decreased in culture over time, with a stepwise increase in the percentage of NSP cells. Even on day 18, cultured NSP cells only contained 0.07±0.03% SP cells (Fig. 4), and these may have arisen from residual SP cells from the last sorting. This suggests that SP cells may undergo asymmetrical cell division to self-renew and generate heterogeneous phenotypes of low-tumorigenic cells, including NSP cells; however, NSP cells cannot reversely differentiate into SP cells.

Neural stem cells have the ability to grow in SFM and form a neurosphere. These properties have been exploited for the selection of stem-like cells in human cancers, represented primarily by breast cancer. In our study, the ability of SP cells to generate spherical colonies was significantly higher than that of NSP cells, consistent with previous studies (Fig. 5A–D) [8], [30].

In the drug sensitivity assay, we measured drug sensitivity differences between SP and NSP cells. The mechanism regulating the efflux of Hoechst dye is conferred in part through the expression of ATP binding cassette protein (ABC) transporters, predominantly ABCG2, involved in the efflux of chemotherapeutic agents [40], [41]. Therefore, we used verapamil to block the membrane ABCG2 transporter and observed the toxicity changes of cisplatin. We found that SP cells were much more resistant to cisplatin than NSP cells at each concentration (P<0.001) except at 11 µg/ml (P>0.05) This situation could be reversed by pretreatment with verapamil, indicating that the ABCG2 transporter may play an important role in drug resistance (Fig. 5E). However, it should be noted that Mdr1a/b/Bcrp1 triple knockout mice are viable and still retain some SP cells in the bone marrow [42], suggesting that the mechanism in which the SP phenotype is determined is not solely conferred through the expression of ABC transporter proteins. Therefore, the exact mechanism of Hoechst dye exclusion remains to be fully elucidated.

Data from independent laboratories, mainly based on the study of cancer cell lines, have demonstrated that when compared to the NSP population, cancer stem-like SP cells are highly enriched for the capacity to initiate tumor formation when xenografted into NOD/SCID mice. The difference in tumorigenic potential between SP and NSP cells varies in different cancer types [31]. In hepatocellular carcinoma cell lines, 1×10^3^ SP cells are sufficient for tumor formation, whereas an injection of 1×10^6^ NSP cells does not initiate tumors. The tumor formation ability of SP cells is 1,000 times higher than that of NSP cells, the most significant difference reported [6]. Importantly, this observation has also been demonstrated in primary mesenchymal tumors, wherein 1×10^2^ SP cells induced tumor formation in 9 mice out of 14; in contrast, 1×10^3^ NSP cells induced tumor formation in 3 mice out of 14. The tumor formation ability of SP cells is about 10 times higher than that of NSP cells [25]. In our study, 2×10^4 ^SP cells were able to induce tumor formation in NOD/SCID mice, whereas 2×10^5^ NSP cells were needed to induce tumor formation. The tumor formation ability of SP cells in the LSCCs is about 10 times higher than that of NSP cells. In addition, significant differences were detected between the weights of SP tumors and NSP tumors (Fig. 6).

In conclusion, our study is the first to demonstrate the existence of cancer stem-like SP cells in purified primary cultured human LSCC epithelial cells. This may serve as a valuable model for CSC research in LSCC. Further studies are needed to identify critical signaling pathways involved in SP cell self-renewal. Targeting these SP cells might result in the development of effective therapeutic agents for LSCC.
